# Delayed-interval delivery can save the second twin: evidence from a systematic review

**Published:** 2016-12

**Authors:** S Feys, Y Jacquemyn

**Affiliations:** Department of Obstetrics and Gynaecology, Antwerp University Hospital (UZA), Edegem, Belgium

**Keywords:** Twin, delivery, dichorionic, tocolysis, cerclage, antibiotics

## Abstract

**Background:**

In case of preterm birth in twins, it is not well established if the second twin benefits from a delayed-interval delivery.

**Objective:**

The main objective of this systematic review is to evaluate survival benefit of the second twin from delayed interval delivery compared to the first twin. Secondly, we will evaluate the survival benefit of the procedure when performed equal to or after 24 weeks gestational age of the first born.

**Methods:**

Delayed interval delivery was defined as every attempt to perform a delayed interval delivery with at minimum placement of a high ligature of the umbilical cord and a delay of delivery of at least 24 hours.

Based on the PRISMA method, a systematic review was performed. Controlled and observational studies reporting at least 3 cases of delayed interval delivery in dichorionic diamniotic twin pregnancy describing the outcome of the first and the second twin were included. Case reports and papers on triplet or higher order pregnancies were excluded.

Primary data included gestational age and outcome of the first and second born. Metadata concern management strategies (tocolysis, antibiotics, cerclage), neonatal data (sex, birth weight and morbidity) and maternal complications.

The methodological quality of included studies was assessed using the "IHE quality appraisal checklist for assessing the quality of case series". Meta-analysis was performed by computing relative risk (RR) with its 95% confidence interval (CI) using the random-effects model. Statistical heterogeneity was tested using the *I^2^* and Chi^2^ statistics.

Since there is no control group for the secondary outcomes, these are presented by narrative synthesis.

**Results:**

Mortality data were extracted from 13 articles, reporting a total of 128 cases of delayed interval delivery. In the analysis, the second born had a significantly lower mortality risk compared to the first born (relative risk = 0.44, 95% confidence interval = 0.34 - 0.57, P<0.0001, I^2^= 0%, P=0.70).

For the analysis of mortality of the second born foetus versus the first born when the first delivery was at ≥24 weeks of gestational age, 12 articles were included. In the analysis 4 reports were excluded since there were no events (no mortality) in both groups (first and second born) making analysis impossible. For the 36 cases included, the second born had a significantly lower mortality risk compared to the first born if delivery of the first born occurred at ≥ 24 weeks gestational age (relative risk=0.37, 95% confidence interval= 0.17 - 0.82, P=0.014, I^2^=0%, P=0.82).

**Conclusions and implications:**

In carefully selected twin pregnancies the survival of the second born twin may improve with delayed interval delivery, also if the first was born at or after 24 weeks. Management protocols in the studies included vary, making it difficult to propose a uniform strategy for delayed interval delivery. Families must be informed about the possibility that a nonviable infant would survive to a periviable gestational age with a risk of severe sequels after birth as well as the possibility of maternal complications.

## Introduction

### Rationale

Twin pregnancies are associated with a higher risk of preterm delivery and this at a significantly earlier gestational age than singletons, resulting in infant morbidity and mortality. (Gardner et al., 1995)

In pregnancies presenting with extremely preterm labour or rupture of the membranes, significant prolongation of gestation and hence increase in foetal weight is expected to improve foetal outcome. For this reason, it can be tried to stop labour after birth of the first foetus. This procedure is defined as a delayed-interval delivery. It was first described by Carson in 1880 with a wait-and-see management (Carson, 1880). Currently, obstetrical management has changed to active management using tocolysis, prophylactic antibiotics and cerclage. Currently delayed interval delivery is defined as every attempt to postpone birth of the second twin with at least placement of a high ligature of the umbilical cord and a delay of delivery of at least 24 hours.

Even though a number of case reports and case series have been published describing attempts to delay delivery of remaining twins, triplets and higher-order multiples after immature delivery, intentional delayed delivery of the second foetus in twin pregnancies is of very rare occurrence. To our knowledge no previous systematic review was performed evaluating the impact of the procedure on the mortality of the second born compared with the first born, nor on neonatal and maternal morbidity.

Furthermore there is no consensus about the effectiveness of the procedure after a specific gestational age. This depends also on procedures relating to neonatal resuscitation on the limits of viability, e.g. in Flanders (Belgium), active management of the new-born is offered from, but not before, 24 weeks of pregnancy, meaning that delaying delivery after 24 weeks highly impacts neonatal treatment. (Jacquemyn et al., 2014)

### Objectives

The main objective of this systematic review is to evaluate survival benefit of the second twin from delayed-interval delivery compared to the first twin. We will review publications describing delayed- interval delivery in dichorionic diamniotic twins. The primary outcome measure is mortality of the first and second twin. When available, full information on gestational age will be registered to complete an additional analysis to evaluate the benefit of the procedure when performed after or equal to 24 weeks gestational age as this is still considered a limit of viability in most high resource countries. Secondary outcomes are neonatal morbidity in first and second twins and maternal morbidity.

## Methods

### Protocol

To ensure the accuracy of this review, we reported it based on the Preferred Reporting Items for Systematic Reviews and Meta-analyses (PRISMA) statement. (Liberati, Altman et al., 2009) Methods of the analysis and inclusion criteria were specified in advance and documented in a review protocol.

## Eligibility criteria

### Study design

Controlled and observational studies were included. Case series were included when reporting at least 3 cases. Case reports were excluded. There was no limit on the year of publication.

### Participants

Only dichorionic diamniotic twin pregnancies were included. Triplet or higher order pregnancies were excluded.

### Interventions

Every attempt to perform a delayed-interval delivery was included since there is no agreement regarding the best management strategy for this procedure. At least high ligature of the umbilical cord should have been performed and a delay of at least 24 hours achieved.

The administration of tocolysis, the use of antibiotics and the placement of cerclage were registered if mentioned in the included article.

### Comparators- outcome

Mortality rate of the first born was compared with the second born. Secondly we examined if there is a difference in mortality between the first and second born if the first twin was born at or after 24 weeks of gestational age.

### Language

Only articles reported in the English and Dutch language were included.

### Information sources

Searching an electronic database identified reports and scanning reference lists of articles. The search was applied to MEDLINE database using PubMed up to November 2014.

### Search

The following terms were used: (all fields) "delayed delivery”, twin AND delayed interval delivery, "interval delivery" AND multiple gestation, delayed interval delivery AND multiple gestation.

### Study selection

Titles were screened first. Secondly, abstracts were read and checked if they accorded with the inclusion criteria. Full reports were obtained for the titles and abstracts that appeared to meet the inclusion criteria, as well as those that were uncertain. Thereafter, the full text reports were screened and decision was made whether these reports met the inclusion criteria.

Constraints were inaccessible full reports. When there were reports using the same database, only the report providing the most applicable information was used.

### Data items

In the excel database following data were registered:

- author and year of publication
- interval (days)
- days of gestation first and second born
- route of delivery of second born (vaginally or caesarean)
- outcome of first and secondborn (APGARscore at 5’)
- tocolysis: product, indication (standard or therapeutic)
- antibiotics: product, indication (standard or therapeutic)
- cerclage: performed or not after delivery of the first twin
- any maternal complications during the period after the first delivery (all information provided)
- neonatal morbidity (all information on foetal morbidity provided)
- foetal sex of the first and second born
- weight of the first and second born

### Risk of bias in individual studies

The methodological quality of included studies was assessed using the IHE quality appraisal checklist for assessing the quality of case series”. For this checklist ‘quality’ covers both risk of bias and quality of reporting. (Guo et al., 2015)

### Summary measures and synthesis of results

Relative risk of mortality was the primary measure of treatment effect. The meta-analysis was performed by computing relative risk (RR) with its 95% confidence interval (CI) using the random-effects model as we expect the interventions in the studies to differ in ways that have impact on the results. Therefore we could not assume a common effect size. (Borenstein et al., 2009) The DerSimonian-Laird method was used for the random effects model.

Statistical heterogeneity was tested using the *I^2^* and Chi^2^ statistics. Heterogeneity was found substantial if an I2 was greater than 30% or if the p-value in the Chi^2^ test for heterogeneity was less than 0.10.

Statistical analysis was carried out using the statistical software StatsDirect 3.0.

### Risk of bias across studies

Funnel plots were used to explore the presence of publication bias. The degree of funnel plot asymmetry was assessed by Begg’s and Egger’s test, p-value with a significant level at 0.05. This statistical analysis was performed using StatsDirect 3.0.

### Additional analysis

Since there is no control group for the secondary outcomes, these results are presented by narrative synthesis.

## Results

### Study selection (Fig. 1)

A total of 13 studies were identified for inclusion, answering our primary research question. For our secondary research question, 12 studies were included. The MEDLINE database search using PubMed and reference lists provided a total of 196 citations. After adjusting for duplicates 103 studies remained. Of these, 76 were discarded because they did not meet the inclusion criteria, based on their abstract (23 abstracts about triplets or higher order multiples, 46 case-reports and seven had no full-text available in English or Dutch). Full text of the remaining 27 citations was examined in detail. Again, 14 reports were discarded. Five because full text of the study was not available. Nine more citations were discarded for different reasons: one concerning a procedure other than delayed- interval delivery, three had a database with many assumptions, one was a review of literature, two examined other outcomes and in two times articles results of database were re-used. No unpublished relevant studies were obtained.

**Fig. 1:**
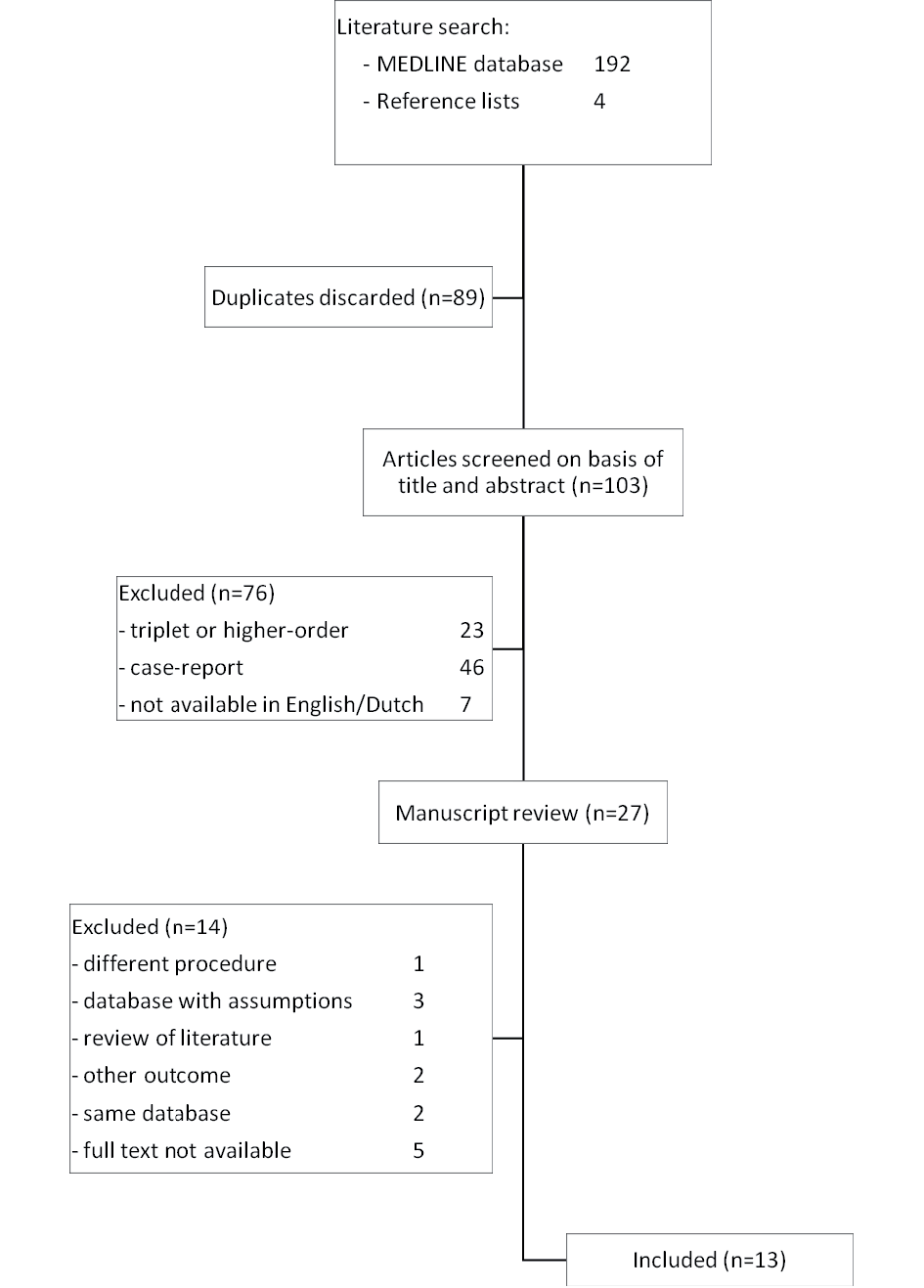
— Flow diagram of study selection

## Characteristics

### Methods

Of the 13 studies finally selected for this review nine were retrospective observational studies (Doger et al., 2014; Padilla-Iserte et al., 2014; Petousis et al., 2012; Arias 1994; Kalchbrenner et al., 1998; van Doorn et al., 1999; Farkouh et al., 2000; Fayad et al., 2003; Cristinelli et al., 2005), three were case series (Wittmann et al., 1992; Van der Straeten et al., 2001; Weemhoff et al., 2001) and one study was a case-control study design (Rosbergen et al., 2005). The retrospective studies described a specific period in which all of the cases with delayed interval delivery registered were reported. One of these retrospective studies was a multicentric study, where an information letter was sent to 20 hospitals enquiring about cases of an interval of more than 48hours. There was response of 12 hospitals. (Fayad et al., 2003) The included case series did not specify a time period in which they registered all cases. This can result in a source of bias where unsuccessful delayed interval delivery intentions are not reported.

The primary outcome of the case-control study was neonatal morbidity compared to a group of singletons born at the same gestational age. (Rosbergen et al., 2005)

### Participants

Within the 13 studies 173 interval delivery procedures were discussed, 132 were twin pregnancies, 33 were triplets, 2 quadruplets and 1 quintuplet. Of the 132 discussed twin pregnancies a total of 128 twins were included. Four were excluded because the interval achieved was <24 hours.

Between studies, the maximum gestational age of the first foetus when delayed-interval delivery was performed, differed. For Kalchbrenner et al. (1998) the delivery of the first foetus should be between 18 and 28 weeks of gestation, for Rosbergen et al. (2005) before 32 weeks of gestation, for Petousis et al. (2012) before 24 weeks of gestation and for Farkouh et al. between 16 and 29 weeks of gestation (Farkouh et al., 2000).

### Intervention

There is no agreement regarding the best management strategy for this procedure. Inclusion criteria for intervention was performing an attempt of delayed-interval delivery with at least high ligature of the umbilical cord being performed and obtaining a delay of at least 24 hours.

There was registration for the use of tocolysis and antibiotics and the placement of cerclage.

Tocolysis was administered prophylactically in 8 reports (Doger et al., 2014; Petousis et al., 2012; Arias, 1994; Kalchbrenner et al., 1998; van Doorn et al., 1999; Farkouh et al., 2000; Van der Straeten et al., 2001; Rosbergen et al., 2005), variably prophylactic or therapeutic use in 3 reports (Wittmann et al., 1992; Weemhoff et al., 2001; Cristinelli et al., 2005), not discussed in one report (Padilla-Iserte et al., 2014) and centre dependent without discussing the modalities in one report (Fayad et al., 2003). Different types of tocolysis were used, depending on local procedures.

In most cases antibiotics were administered prophylactically (Doger et al., 2014; Padilla-Iserte et al., 2014; Arias, 1994; Kalchbrenner et al., 1998; van Doorn et al., 1999; Farkouh et al., 2000; Van der Straeten et al., 2001; Weemhoff et al., 2001; Fayad et al., 2003; Rosbergen et al., 2005). In two reports some patients received antibiotics prophylactically and some therapeutically (Wittmann et al., 1992; Cristinelli et al., 2005) and in one report, antibiotics were only administered in case of preterm premature rupture of membranes (PPROM). When antibiotics were started prophylactically, the duration was very variable. In two reports they were administered for a few days with culture-directed switch according to cervico-vaginal cultures (Padilla-Iserte et al., 2014; Rosbergen et al., 2005). The type of antibiotic therapy was generally a broad-spectrum antibiotic.

In four reports cerclage placement was performed in all cases (Petousis et al., 2012; Arias, 1994; Kalchbrenner et al., 1998; Farkouh et al., 2000). It was dependent on preference of patient and clinician in seven reports (Doger, Cakiroglu et al., 2014; Wittmann et al., 1992; van Doorn et al., 1999; Van der Straeten et al., 2001; Fayad et al., 2003; Cristinelli et al., 2005). In one report it was never placed (Rosbergen et al., 2005) and in one report it was not mentioned (Padilla-Iserte et al., 2014).

## Outcomes

### Primary

In all studies mortality of the first and second born twin was registered. Some studies had a different primary outcome but they were included if data on mortality were mentioned.

Fayad et al did not mention the term of delivery of the first and second baby, for the second analysis this report was excluded. (Fayad et al., 2003)

### Secondary

Only Kalchbrenner et al. specified the maternal complications they would screen for in advance so possibly there is a reporting bias. (Kalchbrenner et al., 1998) Also for neonatal morbidity there was no unambiguous registration. Only Rosbergen et al. (2005) had a clear registration on neonatal morbidity.

For the registration of maternal and neonatal morbidities we used the same reports as for the first analysis with that difference that higher order multiples were also included. The goal of this registration is to mention the possible morbidities rather in a descriptive than a quantitative way, so our results can function as a starting point for future research.

### Risk of bias within studies

The quality of the methodology of included studies was assessed using the "IHE quality appraisal checklist for assessing the quality of case series”.

### Results of individual studies

**Fig. 2:**
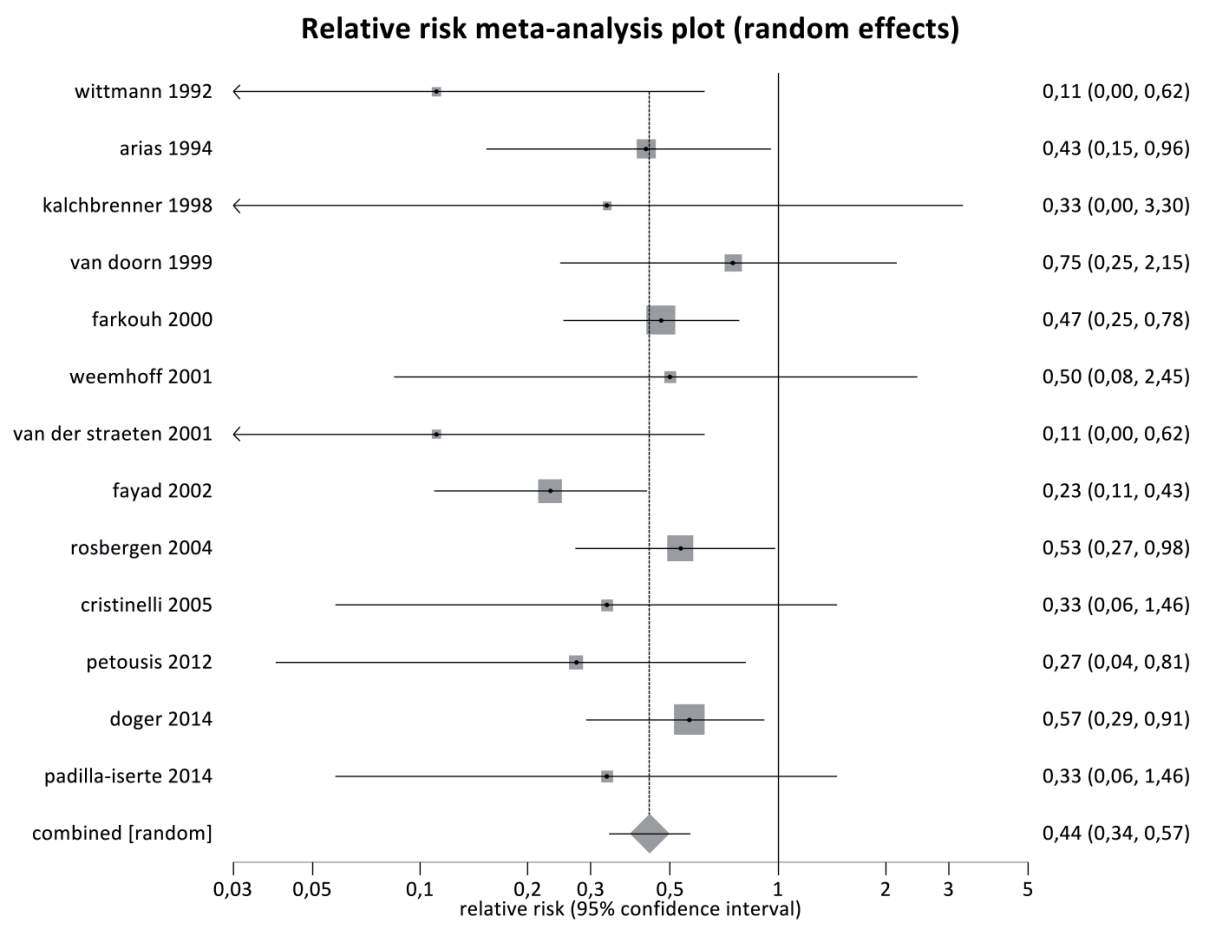
— Forest plot showing relative mortality risk of second born twin after delayed-interval delivery compared to first born twin.

Results of individual studies, relative risk and confidence intervals (CI) for mortality of the second born versus the first born are presented in Table I. Funnel plot is presented in Figure 2.

**Table I:** — Results of individual studies, mortality of the second born versus the first born.

**Study (N, number of twin pregnancie)**	A*	B*	C*	D*	**Relative Risk**	**95% Confidence Interval (CI, Koopman)**		**% Weights (random)**
Wittmann 1992 (4)	0	4	4	0	0.11	0.00	062	0.96
Arias 1994 (8)	3	7	5	1	0.40	0.15	0.96	7.76
Kalchbrenner 1998 (5)	0	1	5	4	0.33	0.00	3.29	0.75
Van Doorn 1999 (7)	3	4	4	3	0.75	0.25	2.15	5.89
Farkouh 2000 (20)	8	17	12	3	0.47	0.25	0.78	20.94
Weemhoff 2001 (3)	1	2	2	1	0.50	0.08	2.45	2.11
Van der Straeten 2001 (5)	0	4	5	1	0.11	0.00	0.62	0.93
Fayad 2002 (28)	6	26	22	2	0.23	0.11	0.43	13.13
Rosbergen 2004 (24)	8	15	16	9	0.53	0.27	0.98	16.21
Cristinelli 2005 (4)	1	3	3	1	0.33	0.06	1.46	2.11
Petousis 2012 (5)	1	5	4	0	0.27	0.04	0.81	3.41
Doger 2014 (11)	6	11	5	0	0.57	0.29	0.91	23.68
Padilla-Iserte 2014 (4)	1	3	3	1	0.33	0.06	1.46	2.11

* A = exposed positive = second born twin with Apgar Score at 5 minutes (AS 5’) = 0 * B = control positive = first born twin with AS 5’ = 0 * C = exposed negative = second born twin with AS 5’ > 0 * D = control negative = first born twin with AS 5’ > 0

**Fig. 3:**
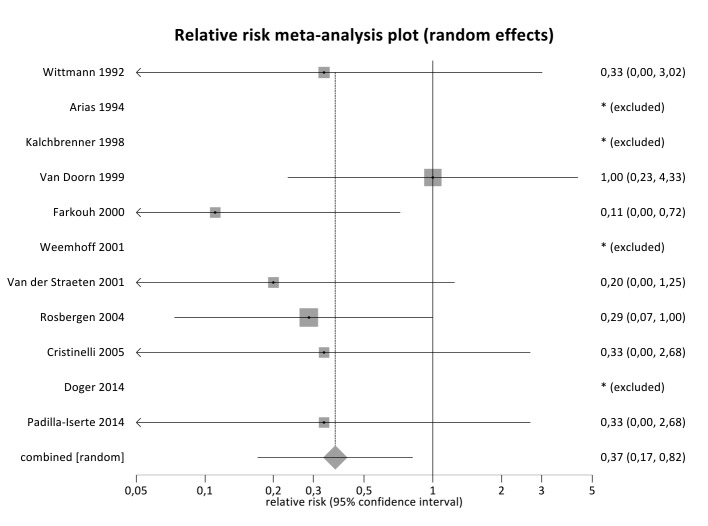
— Forest plot showing relative mortality risk of second born twin after delayed-interval delivery compared to first born twin, with first born delivery at ≥ 24 weeks of gestation.

Results of individual studies, relative risk and confidence intervals for mortality of the second born versus the first born when delivery of the first was ≥ 24 weeks gestational age are presented in Table II. Funnel plot is presented in Figure 3.

**Table II:** — Results of individual studies, mortality of the second born versus the first born, when the first delivery was at ≥24 weeks gestational age.

**Study (N= number of twins in study)**	A*	B*	C*	D*	**Relative Risk**	**95% Confidence Interval (CI, Koopman)**		**% Weights (random)**
Wittmann 1992 (2)	0	1	1	0	0.33	0.00	3,02	9,63
Arias 1994 (1)	0	0	1	1	NA	NA	NA	NA
Kalchbrenner 1998 (4)	0	0	4	4	NA	NA	NA	NA
Van Doorn 1999 (5)	2	2	3	3	1.00	0.23	4.33	26.75
Farkouh 2000 (7)	0	4	7	3	0,11	0	0.72	8.14
Weemhoff 2001 (1)	0	0	1	1	NA	NA	NA	NA
Van der Straeten 2001 (7)	0	2	3	1	0.20	0.00	1.25	8.45
Rosbergen 2004 (18)	2	7	14	9	0.29	0.07	0.99	30.99
Cristinelli 2005 (2)	0	1	2	1	0.33	0.00	2.68	8.02
Doger 2014 (1)	1	1	0	0	NA	NA	NA	NA
Padilla-Iserte 2014 (2)	0	1	2	1	0,33	0.00	2.68	8.02

* A = exposed positive = second born twin with Apgar Score at 5 minutes (AS 5’) = 0 * B = control positive = first born twin with AS 5’ = 0 * C = exposed negative = second born twin with AS 5’ > 0 * D = control negative = first born twin with AS 5’ > 0 NA: Not Applicable

### Synthesis of results

Mortality data were studied in 13 articles, reporting a total of 128 cases of delayed-interval delivery. In the analysis, the second born is associated with significantly lower mortality compared to the first born (relative risk = 0.44, 95% confidence interval = 0.34 – 0.57, p<0.0001). There is no evidence of heterogeneity (*I^2^*=0%, p=0.70).

For the analysis of mortality second born versus first born when the first delivery was ≥24 weeks of gestational age 12 articles were included. In the analysis 4 reports were excluded since there were no events (no mortality) in both groups (first and second born) and the analysis for those reports was not possible. For the 36 cases included, the second born is associated with significantly lower mortality compared to the first born (relative risk=0.37, 95% confidence interval= 0.17 - 0.82, p=0.014). There is no evidence of heterogeneity (*I^2^*=0%, p=0.82).

### Risk of bias across studies

Funnel plot, Begg’s test and Egger’s test were used to explore the publication bias. The funnel plots were symmetrical in general for both analyses.

The Begg’s test and Egger’s test showed no evidence for publication bias in meta-analyses. For the first analysis Begg’s test p=0.11 and Egger’s test p=0.11. For the second analysis Begg’s test p=0.88, Egger’s test p=0.24.

### Additional analysis

Only Kalchbrenner et al. (1998) specified in advance the maternal complications they would screen for. The lack of such research question can cause reporting bias.

There were 4 reports that did not report on maternal morbidity. For those we do not know if there were no complications or if they were just not mentioned. (Petousis et al., 2012; Wittmann et al., 1992; Fayad et al., 2003; Rosbergen et al., 2005) In total, 90 pregnant women were included. There were 28 cases of chorioamnionitis and one was followed by sepsis and septic shock. Placental solutio was mentioned four times with one leading to excessive blood loss of more than 2 litres. In total 3 patients were reported to have severe bleeding. More rare was thromboflebitis that was mentioned in 2 cases (2.2%) and psychological problems in one case. There were no cases of maternal death reported.

As well for neonatal morbidity, systematic registration was lacking in the include studies. In most reports there was no protocol for registration. Only Rosbergen et al. (2005) and Kalchbrenner et al. (1998) had a clearly specified registration strategy on neonatal morbidity. Furthermore Rosbergen et al. used a control group for the second born neonates, to evaluate morbidity due to prematurity or the procedure, concluding the long-term outcome was comparable to children with the same gestational age.

## Discussion

### Summary of evidence

The main objective of this review was to evaluate survival benefit of the second twin from delayed- interval delivery compared to the first twin. A literature search resulted in 13 reports, mainly retrospective case series. A meta-analysis for mortality was performed using data of 128 twins. This analysis showed that the second born has a significantly lower mortality risk compared to the first born (relative risk = 0.44, 95% CI 0.34-0.57, p<0.0001).

Secondly we analysed if there was a benefit if this procedure was performed when the first twin was delivered at or after 24 weeks gestational age. Thirty- six cases were included, showing a significantly lower mortality risk for the second born (relative risk =0.37, 95% CI 0.17-0.82, p=0.014). Although risk of bias analysis using Begg’s and Egger’s test showed no publication bias, results should be interpreted with caution.

For the registration of maternal as well as neonatal morbidity only respectively two and one publication mentioned the reported outcomes in advance. It is difficult to draw conclusions because when a reporting protocol is lacking, the variety of outcomes reported is large. Our goal was to mention the possible morbidities in a descriptive rather than a quantitative way, facilitating future research.

## Limitations

### Outcome level

The meta-analysis reported here combines data across studies in order to estimate treatment effects with more precision than is possible in a single study. A major limitation of this meta-analysis is that the management strategies did not follow a uniform pattern, such as the performance of cerclage, administration of antibiotics and tocolysis. Studies also differed in reporting maternal and neonatal morbidity not using standardized checklists.

### Study level

Publication bias is a potential problem in systematically reviewing case series. Positive results tend to be published more frequently than negative results. In the retrospective case series included in our review, any failed attempts at performing delayed-interval delivery may not have been recorded in the notes, with only the successful attempts recorded. In all but two reports, cases were registered during a period that was specified in advance. This should have minimized publication bias.

### Review level

Limitation on review level was the use of only one online database and restriction to English and Dutch language. Another important remark is the variation in year of publication. This diverges from 1992 to 2014. Since this is a long period it is conceivable that improvement of obstetric and neonatal care can cause different outcome of the included patient group nowadays. There was also an exclusion of unsuccessful procedures since the delay time was set at least 24 hours.

## Conclusion

### Implications for practice

From this review we can conclude that carefully selected twin pregnancies may benefit from delayed-interval delivery as this improves the survival of the second twin. Protocols however vary enormously so there is absence of agreement regarding the best management of these pregnancies. Families must be informed about the possibility that a nonviable infant would survive to a periviable gestational age with a risk of severe sequels after birth as well as the possibility of maternal complications.

### Implications for research

Large multicentre studies should be performed investigating the best management strategy as well as the neonatal and maternal morbidity. These studies should randomize between attempting delayed delivery or not and in case of a delay procedure at least for the use of tocolytics, and preferably also for antibiotics and placement of cerclage. For morbidity investigation a registered study protocol should be used as well as a control group.
